# The epidemiological characteristics and infection risk factors for extrapulmonary tuberculosis in patients hospitalized with pulmonary tuberculosis infection in China from 2017 to 2021

**DOI:** 10.1186/s12879-023-08410-w

**Published:** 2023-09-01

**Authors:** Tianshui Niu, Fei He, Jianshe Yang, Chengxi Ma, Jingyi Xu, Tianzhi Sun, Xin Zhang, Shuyi Chen, Chuhui Ru

**Affiliations:** 1 Department of Pulmonary and Critical Care Medicine Center, Affiliated Hangzhou Chest Hospital, Zhejiang University School of Medicine, Hangzhou, 310058 China; 2 Shanghai Research Center for Thyroid Diseases, Shanghai Tenth People’s Hospital, Tongji University School of Medicine, Shanghai, 200072 China

**Keywords:** Pulmonary tuberculosis, Extrapulmonary tuberculosis, Risk factors, Complication, Disease burden

## Abstract

**Background:**

Pulmonary tuberculosis (PTB) complicated with extrapulmonary tuberculosis (EPTB) infection can aggravate the disease, but there have been few reports.

**Methods:**

Retrospective analysis was used to collect the clinical data of PTB patients with pathogen positive in a teaching hospital from 2017 to 2021. We describe the incidence, the invasive site of EPTB patients, and analyze the infection risk factors for PTB with EPTB by univariate and multivariate logistic regression models. We also compared the complications, disease burden with chi-square test and rank-sum test.

**Results:**

A total of 1806 PTB were included, of which 263 (14.6%) were complicated with EPTB. The common invasive sites for EPTB were neck lymph nodes (16.49%), intestines (16.13%), and meninges (10.75%). Age ≤ 40 (OR = 1.735; 95%CI [1.267–2.376]; *P* = 0.001), malnutrition (OR = 2.029; 95%CI [1.097–3.753]; *P* = 0.022), anemia (OR = 1.739; 95%CI[1.127–2.683]; *P* = 0.012), and osteoporosis (OR = 4.147; 95%CI [1.577–10.905]; *P* = 0.004) were all independent risk factors for PTB infection with EPTB. The incidence of extrathoracic hydrothorax, intestinal bacterial infection, urinary tract bacterial infection, and abdominal bacterial infection were higher in patients with PTB with EPTB. PTB with EPTB patients also had longer median hospitalization durations (19 vs. 14 days), during which time they incurred higher total costs, laboratory test costs, imaging examination costs, and drug use costs.

**Conclusion:**

This study found important risk factors for PTB complicated with EPTB, such as age ≤ 40, malnutrition, anemia, and osteoporosis. PTB with EPTB patients have more extrapulmonary complications and higher hospitalization disease burden.

## Introduction

Tuberculosis is an ancient disease and is still an important cause of death worldwide. According to recent statistics, approximately 25% of the world’s population is infected with *Mycobacterium tuberculosis* [1]. During the ongoing COVID-19 pandemic, the number of patients infected and dying of tuberculosis, globally, has increased, reversing the declining trend over recent years. The World Health Organization (WHO) global tuberculosis report 2022 identified that in 2021, there were about 10.6 million new tuberculosis patients, and 1.6 million people died of tuberculosis [2]. Thus, tuberculosis poses a serious threat to global public health and remains an important public health problem worldwide.

Compared with the widespread concern associated with pulmonary tuberculosis (PTB), extrapulmonary tuberculosis (EPTB) has not received much attention. EPTB refers to infection of organs and body sites outside the lung, trachea, bronchus, and pleura, by *Mycobacterium tuberculosis*. EPTB can affect almost any organ and results in atypical clinical symptoms and atypical imaging findings. In addition, the difficulty in acquiring samples and the low etiological positive rate make the effective diagnosis and management of EPTB challenging [3–5]. Compared with PTB, the increase of EPTB is more significant. The 2020 global tuberculosis report states that 16% of all tuberculosis patients, in 2019, presented also EPTB[6]. In the United States and Europe, the proportion of EPTB cases is between 20% and 53% [7–9]. Since EPTB is neither a notifiable disease nor routinely managed, in China, it is frequently misdiagnosed or missed. Consequently, a large number of EPTB patients are excluded from public health reports. One study reported that 33.4% of TB inpatients in Beijing are EPTB [10].

The proportion of PTB patients with EPTB is also high. In the United States, between 1993 and 2006, the proportion of PTB patients with EPTB was 6% [11]. The results of multicenter research in China from 2011 to 2017 showed that the proportion of PTB patients with EPTB was 11.93% [12]. Although PTB complicated with EPTB infection can aggravate the disease, prolong the course of treatment, and increase the disease burden, there have been few reports on PTB patients with EPTB. A greater understanding of the epidemic characteristics and risk factors associated with PTB with EPTB should play an important role in formulating corresponding prevention and control measures. Thus, in this study, we analyze the epidemiological characteristics, risk factors, complications, and economic burden of PTB patients complicated with EPTB, to provide reference data for the early detection and treatment of PTB patients complicated with EPTB.

## Materials and methods

### Data sources

Single-institution, retrospective analysis was used to collect the case information of pathogen-positive patients admitted to the Hangzhou Chest Hospital from January 1, 2017 to December 31, 2021. This study was approved (approval number 2021 − 232) by the Ethics Committee of Hangzhou Thoracic Hospital.

The case inclusion criteria were as follows: culture positive (solid or liquid sputum culture) for *Mycobacterium tuberculosis*; and rifampicin drug sensitivity data for isolate determined by either a drug sensitivity test or GeneXpertMTB/RIF detection of *Mycobacterium tuberculosis* DNA. The following exclusion criteria were applied: (1) cases with no etiological results, sputum smear positive only, aseptic identification or absence of rifampicin drug sensitivity data, (2) patients with hospital stays shorter than 2 days, (3) cases with incomplete clinical data. The case information on first admission was used for patients hospitalized multiple times. Tuberculous pleurisy and bronchial tuberculosis are classified as pulmonary tuberculosis by the national diagnostic criteria of pulmonary tuberculosis (WS288-2017) [13]. The definition and classification of EPTB were carried out in accordance with the Chinese national guidelines for the diagnosis of EPTB [14].

### Date collection

Clinical data and laboratory results were collected from the hospital information system (HIS). The following data were collected: (1) demographic parameters, including the sex, age, habitation, and occupation of the patient; (2) history of tuberculosis treatment; (3) sputum smear results; (4) *Mycobacterium tuberculosis* rifampicin drug sensitivity; (5) ICU admission; (6) underlying diseases, (7) Body infected sites by EPTB; (8) complications in hospital; (9) length of hospital stay; (10) and economic burden, including the total cost of hospitalization, laboratory testing costs, imaging examination costs, and drug use costs.

### Method process

**Fig. 1:**
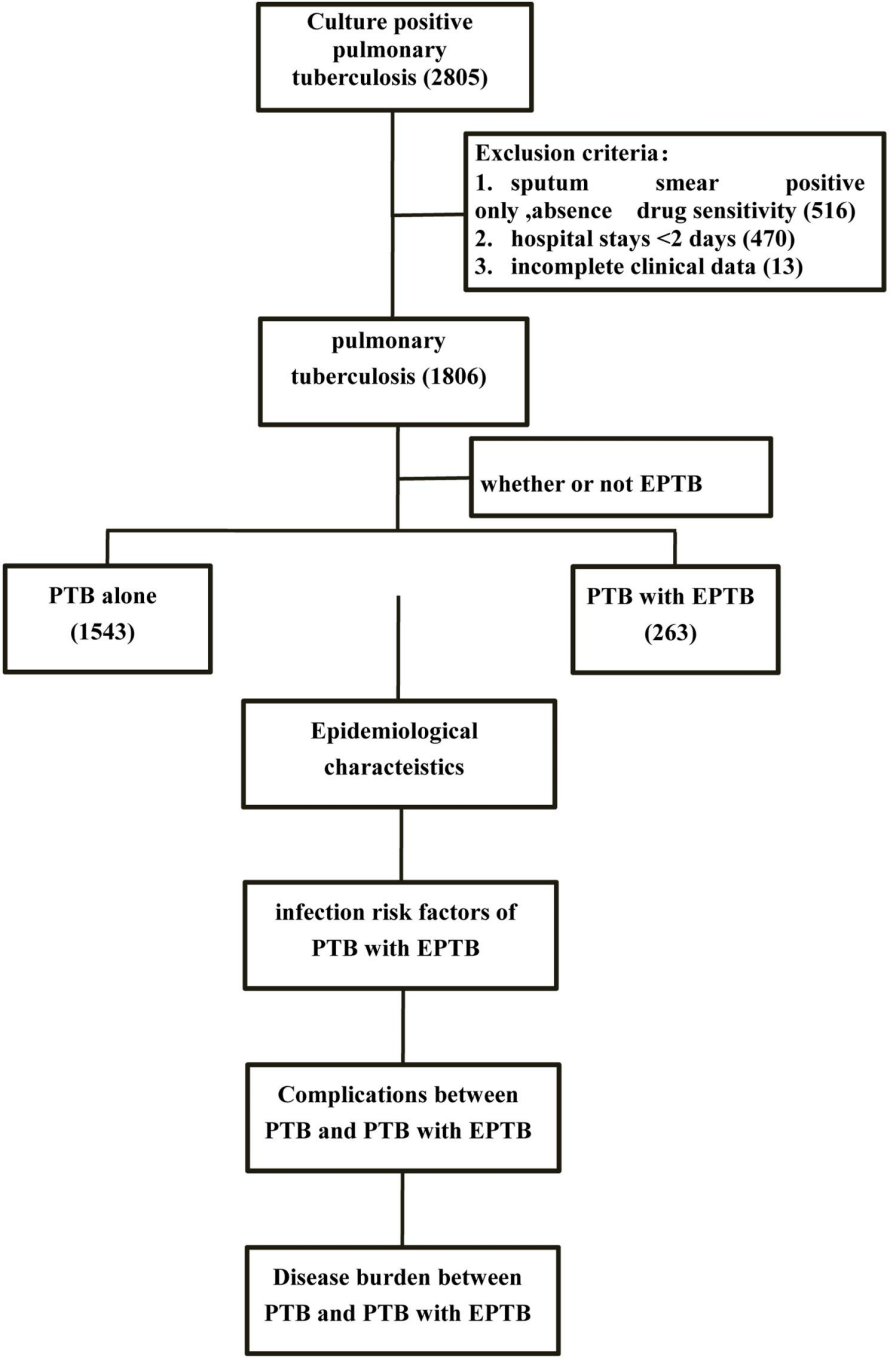
Case selection process

Our research process was shown in Fig. 1, and the specific process was as follows: according to the exclusion criteria, the final included patients were divided into patients with PTB alone and PTB with EPTB according to whether they have EPTB. We described the epidemiological characteristics of pulmonary tuberculosis patients. We used univariate and multivariate logistic regression to analyze the risk factors of PTB with EPTB infection, and used chi-square to compare the complications of. We analyzed the disease burden of burdern PTB alone and PTB with EPTB using rank-sum analysis.

### Statistical analysis

SPSS 23.0 was used for statistical analysis. The chi-squared and Fisher’s exact tests were used to analyze categorical variables. The *t*-test and Wilcoxon rank-sum test were used to analyze continuous variables. Variables with a *P*-value < 0.10 in the univariate analysis were entered into a multivariate analysis using the multiple logistic regression method to determine the independent variables that were associated with PTB with EPTB infection. Variables with a *P*-value < 0.05 were considered independent risk factors, and the results are presented as the odds ratio (OR) and 95% confidence intervals (95% CI). One-way ANOVA was used to analyze the length of hospital stay and economic burden of disease during hospitalization. All tests were two-tailed, and a *P*-value < 0.05 was considered significant.

## Results

### Patient characteristics

The application of inclusion/exclusion criteria to HIS data identified 1806 patients hospitalized with pulmonary tuberculosis, from 2017 to 2021, suitable for inclusion in this study; 1543 (85.44%) with PTB and 263 (14.56%) with EPTB. Of the study cohort, 1238 (68.55%) were males, and 568 (31.45%) were female; 52 patients were readmitted within 2 months, and 186 patients were admitted multiple times within 1 year. The median age of the cohort was 55 years old; the youngest patient was 5 years old, and the oldest was 97 years old. 593 (32.83%) cases were less than 40 years old, and 1213 (67.17%) cases were more than 40 years old. The cohort comprised 928 (51.38%) patients from rural areas and 878 (48.62%) from urban areas. The cohort included 587 (32.50%) farmers, 94 (5.20%) workers, 85 (4.71%) students, 133 (7.36%) unemployed people, 213 (11.79%) employees, 258 (14.29%) retirees and 436 (24.14%) Other jobs. Ninety-five (5.26%) of the cohort were admitted to ICU. There were 994 (55.04%) sputum smear-positive patients, 1463 (81.01%) newly treated patients, and 343 (19.00%) retreated patients. The *Mycobacterium tuberculosis* isolates from 781 (43.24%) patients were resistant to rifampicin. Among the underlying diseases, hypertension was found in 329 (18.22%) patients, diabetes in 313 (17.33%), coronary in 233 (12.90%), anemia in 163 (9.03%), and chronic obstructive pulmonary disease/chronic bronchitis in 156 (8.64%). The most common complications during hospitalization were pulmonary infections (675 cases; 37.38%), medicated hepatitis (354 cases; 19.60%), hypoproteinemia (274 cases; 15.17%), and respiratory failure (160 cases; 8.86%). The median length of stay was 14 days, the median total cost during hospitalization was 1.75 × 10^4^ Renminbi (RMB), the median cost of laboratory testing was 3.79 × 10^3^ RMB, the median cost of imaging examination was 4.15 × 10^2^ RMB, and the median cost of drugs was 0.49 × 10^4^ RMB.

### Body infected sites by EPTB

The body sites most commonly infected by EPTB were neck lymph nodes (46 cases; 16.49%), intestines (45 cases; 16.13%), meninges (30 cases; 10.75%), throat (26 cases; 9.32%), and thoracic vertebrae (23 cases; 8.24%). Based on the classification of system and function, the most common sites of infection were the lymphatic system (79 cases; 28.32%), digestive system (64 cases; 22.93%), motor system (58 cases; 20.79%), head and facial features (56 cases; 20.07%), and urogenital system (13 cases; 4.66%: Table 1).

**Table 1:** Tissues or organs infected by EPTB

Tissue or organ infected by EPTB	Number of infringements^a^	Complication rate (%)^b^	Constituent ratio (%)^c^
Lymph gland	79	4.37	28.32
neck	46	2.55	16.49
mediastinum	22	1.22	7.89
armpit	5	0.28	1.79
mesentery	2	0.11	0.72
abdominal cavity	4	0.22	1.43
Head and facial features	56	3.10	20.07
meninges	30	1.66	10.75
throat	26	1.44	9.32
Digestive system	64	3.54	22.93
abdomen	14	0.78	5.01
intestine	45	2.49	16.13
Liver and spleen	7	0.39	2.51
Perianal	1	0.06	0.36
Circulatory system	9	0.50	3.23
pericardium	9	0.50	3.23
Motor system	58	3.21	20.79
spine	2	0.11	0.72
thoracic vertebra	23	1.27	8.24
lumbar vertebra	12	0.66	4.30
bone	2	0.11	0.72
joint	19	1.05	6.81
Urogenital system	13	0.72	4.66
Kidney, adrenal gland, bladder	5	0.28	1.79
pelvic cavity	3	0.17	1.08
Male reproductive system (testis, epididymis, prostate)	4	0.22	1.43
breast	1	0.06	0.36
Total	279	15.45	100.0

Footnotes: ^a^Where complications of EPTB infected multiple organs or tissues of the same patient, the invasion frequency was calculated according to the number of sites infected. Thus, the total infection number (279) is greater than the total number of patients with EPTB (263 cases); ^b^Complication rate = infringement number/number of pulmonary tuberculosis patients investigated (1806 cases); ^c^Constituent ratio = infringement number/total infringement number (279) × 100

### Analysis of risk factors associated with EPTB

Univariate analysis showed that gender (female), age ≤ 40, rural habitation, unemployed, sputum smear positive, rifampicin resistance, COPD/chronic bronchitis, Bronchiectasia, Hypertension, Coronary, Diabetes, Malnutrition, Anemia, and Osteoporosis were significantly associated with PTB with EPTB infection. Multivariate regression analysis showed that age ≤ 40 (OR [95% CI] = 1.735 [1.267–2.376]; *P* = 0.001), malnutrition (OR [95% CI] = 2.029 [1.097–3.753]; *P* = 0.024), anemia (OR [95% CI] = 1.739 [1.127–2.683]; *P* = 0.012), and osteoporosis (OR [95% CI] = 4.147 [1.577–10.905]; *P* = 0.004) are independent risk factors for PTB with EPTB infection. By contrast, positive sputum smear (OR [95% CI] = 0.738 [0.560–0.973]; *P* = 0.031), *Mycobacterium tuberculosis* rifampicin resistance (OR [95% CI] = 0.681 [0.515–0.900]; *P* = 0.007), bronchiectasis (OR [95% CI] = 0.326 [0.117–0.910]; *P* = 0.032), and diabetes mellitus (OR [95% CI] = 0.568 [0.357–0.904]; *P* = 0.017) are protective factors for PTB with EPTB infection (Table 2).

**Table 2:** Epidemiological characteistics and infection risk factors of pathogen positive pulmonary tuberculosis (PTB) with extrapulmonary tuberculosis (EPTB)

Risk factor, n (%)	PTB alone (n = 1543)	PTB with EPTB (n = 263)	Univariate analysis, P value	Multivariate logistic regression analysis	
				OR (95% CI)	P value
Sex					
Male	1071(69.41)	167(63.50)	-		
Female	472(30.59)	96(36.50)	0.034	1.058 (0.792–1.413)	0.702
Readmission within 60 days					
No	1495(96.89)	259(98.48)	-		
YES	48(3.11)	4(1.52)	0.104		
More than 2 hospitalizations within 1 year					
No	1386(89.83)	234(88.97)	-		
YES	157(10.17)	29(11.03)	0.371		
Age group, y					
≤ 40	469 (30.40)	124 (47.15)	0.000	1.735 (1.267–2.376)	0.001
> 40	1074(69.60)	139(52.85)	-	-	
Residence					
Urban	764(49.51)	114 (43.35)	-		
Rural	779(50.49)	149(56.65)	0.037	1.175 (0.885–1.561)	0.264
Profession					
Farmer	510(33.05)	77(29.28)	0.127		
Worker	83(5.38)	11(4.18)	0.262		
Student	68(4.41)	17(6.46)	0.100		
Unemployed	105(6.80)	28(10.65)	0.022	1.313 (0.826–2.089)	0.250
Staff member	179(11.60)	34(12.93)	0.299		
Retiree	228(14.78)	30(11.41)	0.086	1.184 (0.748–1.875)	0.471
Other	370(23.97)	66(25.10)	0.148		
ICU Admission					
No	1463(94.82)	248(94.30)	-		
Yes	80(5.18)	15(5.70)	0.409		
Sputum smear positive					
Negative	671 (43.49)	141 (53.61)			
Positive	872 (56.51)	122 (46.39)	0.001	0.738 (0.560–0.973)	0.031
Treatment history					
New case	1243(80.56)	220(83.65)	0.136		
Retreated case	300(19.44)	43(16.35)	-		
Rifampicin resistance					
No	860(55.74)	165(62.74)			
Yes	683(44.26)	98(37.26)	0.020	0.681 (0.515-0.900)	0.007
Underlying disease					
COPD/chronic bronchitis	146 (9.46)	10 (3.80)	0.001	0.507 (0.253–1.014)	0.055
Bronchiectasia	81 (5.25)	4 (1.52)	0.003	0.326 (0.117–0.910)	0.032
Hypertension	299 (19.38)	30 (11.41)	0.001	0.764 (0.489–1.195)	0.238
Coronary	210 (13.61)	23 (8.75)	0.016	0.778 (0.470–1.290)	0.331
Cerebrovascular	99 (6.42)	20 (7.60)	0.274		
Gastrointestinal diseases	106 (6.87)	15 (5.70)	0.292		
Renal insufficiency	107 (6.93)	21 (7.98)	0.307		
Tumor	95 (6.16)	11 (4.18)	0.130		
Diabetes	289 (18.73)	24 (9.13)	0.000	0.568 (0.357–0.904)	0.017
Malnutrition	54 (3.50)	17 (6.46)	0.022	2.029 (1.097–3.753)	0.024
Anemia	128 (8.30)	35 (13.31)	0.008	1.739 (1.127–2.683)	0.012
Arthrolithiasis	46 (2.98)	9 (3.42)	0.408		
Osteoporosis	15 (0.97)	7 (2.66)	0.031	4.147 (1.577–10.905)	0.004
Arthritis	32 (2.07)	5 (1.90)	0.543		
Prostatic hyperplasia	90 (5.83)	9 (3.42)	0.069	0.954 (0.459–1.982)	0.899
Alcoholic Liver Disease	20 (1.30)	2 (0.76)	0.358		
hepatitis/cirrhosis	100 (6.48)	16 (6.08)	0.469		
Syphilis	7 (0.45)	2 (0.76)	0.385		
AIDS	4 (0.26)	1 (0.38)	0.545		

Data are expressed as numbers (%) unless otherwise stated; Abbreviations: OR, odds ratio; CI, confidence interval; ICU, intensive care unit; COPD, chronic obstructive pulmonary disease; AIDS, Acquired Immune Deficiency Syndrome

### Analysis of complications and disease burden associated with EPTB

Compared with PTB patients, PTB with EPTB patients had a higher incidence of extrathoracic effusion (2.72% vs. 7.98%; *P* = 0.000), intestinal infection (0.13% vs. 3.80%; *P* = 0.000), urinary tract infection (0.71% vs. 2.28%; *P* = 0.027), and abdominal infection (0% vs. 0.76%; *P* = 0.021), and lower incidence of hemoptysis (4.41% vs. 1.52%; *P* = 0.014) (Table 3). The median hospitalization duration of PTB with EPTB patients was longer (19 vs. 14 days; *P* = 0.005), and the median total cost of hospitalization (2.09 × 10^4^ vs.1.68 × 10^4^ RMB; *P* = 0.000), median laboratory diagnosis cost (0.44 × 10^4^ vs. 0.37 × 10^4^ RMB; *P* = 0.000), median image diagnosis cost (4.95 × 10^2^ vs. 4.15 × 10^2^ RMB; *P* = 0.000), and median drug cost during hospitalization (0.61 × 10^4^ vs. 0.47 × 10^4^ RMB; *P* = 0.000) were all significantly higher for PTB with EPTB patients than for PTB patients (Table 4).

**Table 3:** Complications of pathogen positive pulmonary tuberculosis (PTB) with extrapulmonary tuberculosis (EPTB)

Comorbid conditions, n (%)	PTB alone (n = 1543)	PTB with EPTB (n = 263)	Univariate analysis, P value
Pulmonary infection	588 (38.11)	87 (33.08)	0.068
Severe pneumonia	47 (3.05)	8 (3.04)	0.592
Hemoptysis	68 (4.41)	4 (1.52)	0.014
Aerothorax	31 (2.01)	8 (3.04)	0.197
Respiratory failure	139 (9.01)	21 (7.98)	0.343
Pyothorax	11 (0.71)	2 (0.76)	0.585
Pleural effusion	113 (7.32)	17 (6.46)	0.365
Extrathoracic effusion	42 (2.72)	21 (7.98)	0.000
Intestinal obstruction	2 (0.13)	10 (3.80)	0.000
Intestinal infection	2 (0.13)	10 (3.80)	0.000
Urinary tract infection	11 (0.71)	6 (2.28)	0.027
Abdominal infection	0 (0.00)	2 (0.76)	0.021
Mycotic infection	34 (2.20)	5 (1.90)	0.488
Septic shock	17 (1.10)	3 (1.14)	0.575
Electrolyte disorder	125 (8.10)	26 (9.88)	0.197
Acute renal insufficiency	20 (1.30)	6 (2.28)	0.166
Acid-base disturbance	8 (0.52)	0 (0.00)	0.283
Pain	7 (0.45)	1 (0.38)	0.671
Hypoproteinemia	223 (14.45)	51 (19.39)	0.027
Medicated hepatitis	297 (19.25)	57 (21.67)	0.202
Medicated dermatitis	34 (2.20)	6 (2.28)	0.538
Leukocytopenia	136 (8.81)	20 (7.60)	0.305

**Table 4:** Disease burden of pathogen positive pulmonary tuberculosis (PTB) with extrapulmonary tuberculosis (EPTB)

Disease burden	PTB alone (n = 1543)	PTB with EPTB (n = 263)	Univariate analysis, P value
Median length of hospital stay	14(2-328)	19(2-200)	0.005
Median cost in hospitalization (¥)	1.68 × 10^4^(0.15 × 10^4^-57.67 × 10^4^)	2.09 × 10^4^(0.26 × 10^4^-65.58 × 10^4^)	0.000
Median Laboratory diagnosis cost (¥)	0.37 × 10^4^(0.06 × 10^4^- 5.74 × 10^4^)	0.44 × 10^4^(0.01 × 10^4^- 6.23 × 10^4^)	0.000
Median Imaging diagnosis cost (¥)	4.15 × 10^2^(0.00 × 10^2^- 59.98 × 10^2^)	4.95 × 10^2^(0.00 × 10^2^- 69.81 × 10^2^)	0.000
Mediandrug cost in hospitalization (¥)	0.47 × 10^4^(0.02 × 10^4^- 25.14 × 10^4^)	0.61 × 10^4^(0.17 × 10^4^- 34.40 × 10^4^)	0.000

## Discussion

We conducted a detailed analysis of the epidemiological features, clinical characteristics, infection risk factors, and disease burden of patients presenting with PTB and PTB with EPTB at a hospital in southern China from 2017 to 2021. Of the 1806 cases of pathogen-positive PTB we analyzed, 263 (14.56%) cases were PTB with EPTB. This is a higher proportion of PTB with EPTB cases than the 6.1% cases reported in a study of PTB patients presenting at the Beijing Chest Hospital from 2008 to 2017 [10] and the 11.93% cases reported in a multicenter study in China from 2011 to 2017 [12]. By contrast, the proportion of PTB with EPTB cases reported in a study from Hunan Chest Hospital in 2016 was higher at 22.7% [15]. These differences may reflect different sources of disease in different regions and hospitals. All these data show, however, that patients with PTB with EPTB account for a significant proportion of the total PTB patients, and this proportion is likely to increase year by year. EPTB is often overlooked because the clinical manifestations of EPTB are diverse, the symptoms are not typical, and the etiology is difficult to obtain. The routine screening of patients with PTB for EPTB infection, in clinical practice, would significantly improve the effectiveness of treatment of such patients and thus their prognosis.

The site of EPTB infection may vary from region to region or hospital to hospital. In the United States, lymphoid TB (of which more than 60% of cases are neck lymphoid TB) is the most prevalent form, followed by pleural TB [11]. In Germany, the most common forms of EPTB are lymphoid TB (47%), pleural TB (16%), and genitourinary TB (10%)[16]. In the UK, the most common forms of EPTB are lymphoid TB (49%), pleural TB (10%), and gastrointestinal TB (7%) [16]. In Korea, lymph node TB (28.3%) is the main form, followed by abdominal TB (18.4%) and disseminated TB (14.5%) [17]. A multicenter study in China showed that the most common cases were pleurisy TB (50.15%), bronchial TB (14.96%), neck lymph node TB (7.24%), meningitis TB (7.23%), and peritonitis TB (4.79%) [12]. According to the new diagnostic standard in China, pleurisy TB and bronchial TB are included in pulmonary TB. We showed that the most common sites for EPTB are neck lymph nodes (16.49%), intestines (16.13%), and meninges (10.75%), which is in line with the results of these previous studies. In our study, intestinal TB represented a higher proportion of EPTB cases than in the previous studies. Intestinal TB is caused by the invasion of the intestinal tract by *Mycobacterium tuberculosis*, and usually occurs in young and middle-aged women under 40 years of age. This is consistent with our analysis that identified age (≤ 40) and gender (female) as risk factors for EPTB.

A number of studies have reported that young people [10, 18] and females are high-risk populations for EPTB [11, 19, 20]. In our study, we also found that young women are a high-risk population for PTB combined with EPTB. The proportion of PTB cases complicated with EPTB infection was significantly higher than that of PTB alone for both females (36.50% vs. 30.59%; *P* = 0.034) and patients of age ≤ 40 (47.15% vs. 30.40%; *P* < 0.001). Our multivariate analysis also found that malnutrition (OR [95% CI] = 2.029 [1.097–3.753]; *P* = 0.024), anemia (OR [95% CI] = 1.739 [1.127–2.683]; *P* = 0.012), and osteoporosis (OR [95% CI] = 4.147 [1.577–10.905]; *P* = 0.004) were also risk factors for PTB with EPTB infection. By contrast, bronchiectasis (OR [95% CI] = 0.326 [0.117–0.910]; *P* = 0.032) reduced the risk of PTB complicated with EPTB. This suggests that malnutrition, anemia, and osteoporosis caused by systemic nutritional deficiency are important factors in the occurrence of PTB with EPTB infection. Anemia and malnutrition are common underlying diseases of tuberculosis [21–23] and are associated with complications in children with EPTB [24]. Our study also showed that the incidence of anemia and malnutrition in PTB with EPTB patients was higher than that in PTB without EPTB patients. We also showed, for the first time, that osteoporosis is an independent risk factor for PTB with EPTB. We speculate that this association may be related to the following aspects: (1) the use of corticosteroids. In China, patients with PTB with EPTB are likely to use corticosteroids frequently since the medical consensus on the treatment of TB recommends the use of hormones, when necessary, for meningitis TB, pericarditis TB, peritonitis TB, and acute hematogenous disseminated PTB. This may lead to the extensive use of hormones in patients with PTB complicated with EPTB and result in osteoporosis in these patients [25]; (2) direct infiltration of *Mycobacterium tuberculosis* into the bone tissue and a reduction in bone strength. For example, bone nodule complication of TB results in destruction of the soft tissue around the bone, when *Mycobacterium tuberculosis* invades the skeletal system, weakening the maintenance of bone tissue [26]; (3) vitamin D deficiency. Vitamin D deficiency has been confirmed to be an independent risk factor for EPTB [27, 28]. Vitamin D deficiency can lead to the occurrence and deterioration of osteoporosis; (4) malnutrition. Our study found that malnutrition is a risk factor for PTB with EPTB, and malnutrition can lead to the development of osteoporosis [29]. Bronchiectasis and diabetes appear to protect against the development of EPTB following PTB [10, 30–33]. Our data also confirms that EPTB is more likely to occur without diabetes. The lower incidence of EPTB development in patients with diabetes may be related to the following aspects: (1) age. In this study, it was shown that the proportion of patients who developed EPTB following PTB was significantly increased for patients younger than 40 years of age. By contrast, older people have an increased risk of developing diabetes [34]. In other words, diabetic patients are much more likely to be of an age that makes them statistically less likely to develop EPTB; (2) hyperglycemia, which can change the permeability of pulmonary vascular endothelial cells, induce the production of vascular inflammatory factors, promote remodeling of pulmonary vessels, and increase pulmonary hypertension. These consequences of hyperglycemia can result in a decline in respiratory function and in damage to lung defense mechanisms. Thus, diabetic patients are more vulnerable to respiratory diseases, particularly lower respiratory tract infections by atypical pathogens that are associated with more severe attacks of pneumonia [35–37]; (3) impaired immune response. Patients with diabetes are more likely to have an impaired immune response, which promotes the primary TB infection or reactivation of latent TB [38].

We observed that patients with PTB with EPTB had complications outside the lungs, including extrathoracic effusion, intestinal infection, urinary tract infection, and abdominal infection. Therefore, PTB combined with EPTB is more harmful to patients. 

Tuberculosis is a high-burden disease, and studies from the United States show that the total cost of hospitalization is $16,695 per TB case. Indeed, the total TB-related hospitalization costs in the United States, in 2014, were estimated at $123.4 million [39]. Our in-depth research on the burden of disease, in China, found that the duration of hospitalization of PTB with EPTB patients was significantly prolonged (19 days vs. 14 days), and the total cost, laboratory testing cost, imaging examination cost, and drug use cost of PTB with EPTB patients during hospitalization were also significantly higher. This is consistent with other report, which showed that EPTB and combined PTB are associated with longer hospital stays (11.27 vs. 8.56 days) and higher financial burden rates (17.67% vs. 13.30%) than PTB alone [40].

In summary, PTB with EPTB is more harmful than PTB alone; however, the diverse clinical manifestations of EPTB infection make its diagnosis more difficult. Consequently, the identification of robust risk factors associated with EPTB would allow patients with PTB to be screened to facilitate diagnosis, monitoring, and management of individuals at higher risk of developing EPTB. In turn, this should reduce the hospitalization time of patients and reduce the financial burden of the disease. 

This study also has the following limitations: (1) this is a retrospective study in which the data analyzed were from patients presenting at a single center. Since our hospital is a designated hospital for tuberculosis, with a high proportion of critically ill patients, there may be some selection bias, and the sample may not be widely representative; (2) for this study, we selected only those PTB patients who returned a positive bacterial culture. Consequently, the characteristics of patients with a clinical diagnosis of PTB or PTB with EPTB, but who did not give a positive culture result, were not included. This may also have introduced some bias. 

## Conclusion

In summary, we found important risk factors for PTB complicated with EPTB, such as age ≤ 40, malnutrition, anemia, and osteoporosis. PTB with EPTB patients have more extrapulmonary complications and higher hospitalization disease burden. The clinical characteristics, epidemiological characteristics, risk factors, complications, and disease burden of this patient cohort provide important reference data for the diagnosis and treatment of PTB complicated with EPTB.

## Data Availability

The datasets used and/or analyzed during the current study are available from the corresponding author on reasonable request.

## References

[1] Koegelenberg CFN, Schoch OD, Lange C. Tuberculosis: the past, the Present and the Future.Respiration. 2021;100(7):553–6. 10.1159/000516509 34034257

[2] World Health Organization. Global Tuberculosis Report 2022.

[3] Sharma SK, Mohan A, Kohli M (2021). Extrapulmonary tuberculosis. Expert Rev Respir Med.

[4] Rodriguez-Takeuchi SY, Renjifo ME, Medina FJ (2019). Extrapulmonary Tuberculosis: Pathophysiology and Imaging Findings Radiographics.

[5] Walzl G, McNerney R, du Plessis N (2018). Tuberculosis: advances and challenges in development of new diagnostics and biomarkers. Lancet Infect Dis.

[6] World Health Organization. Global tuberculosis report 2020.

[7] Rowińska-Zakrzewska E, Korzeniewska-Koseła M, Roszkowski-Śliż K (2013). Extrapulmonary Tuberculosis in Poland in the years 1974–2010. Pneumonol Alergol Pol.

[8] Sandgren A, Hollo V, van der Werf MJ (2013). Extrapulmonary tuberculosis in the European Union and European Economic Area, 2002 to 2011. Euro Surveill.

[9] Kruijshaar ME, Abubakar I (2009). Increase in extrapulmonary tuberculosis in England and Wales 1999–2006. Thorax.

[10] Pang Y, An J, Shu W et al. Epidemiology of Extrapulmonary Tuberculosis among Inpatients, China, 2008–2017.Emerg Infect Dis. 2019;25(3):457–64. 10.3201/eid2503.180572 PMC6390737 30789144

[11] Peto HM, Pratt RH, Harrington TA (2009). Epidemiology of Extrapulmonary Tuberculosis in the United States, 1993–2006. Clin Infect Dis.

[12] Kang W, Yu J, Du J (2020). The epidemiology of extrapulmonary tuberculosis in China: a large-scale multi-center observational study, the epidemiology of extrapulmonary tuberculosis in China: a large-scale multi-center observational study. PLoS ONE.

[13] Diagnosis for pulmonary tuberculosis (2018). Chin J Infect Control.

[14] Ma Y, Zhu L, Pan Y (2006). Tuberculosis. [in Chinese].

[15] Xu Z, Liu L, Wang Q (2021). Study on epidemiological characteristics and influencing factors of pathogen positive hospitalized pulmonary tuberculosis patients with extrapulmonary tuberculosis. Chin J Antituberculosis.

[16] Solovic I, Jonsson J, Korzeniewska-Koseła M (2013). Challenges in diagnosing extrapulmonary tuberculosis in the European Union, 2011. Euro Surveill.

[17] Lee MK, Moon C, Lee MJ (2021). Risk factors for the delayed diagnosis of extrapulmonary TB. Int J Tuberc Lung Dis.

[18] Wang DM, Li QF, Zhu M (2017). Analysis of infection and drug-resistance in 6107 cases of extrapulmonary tuberculosis in Chengdu area. Zhonghua Jie He He Hu Xi Za Zhi.

[19] Sunnetcioglu A, Sunnetcioglu M, Binici I (2015). Comparative analysis of pulmonary and extrapulmonary tuberculosis of 411 cases. Ann Clin Microbiol Antimicrob.

[20] Forssbohm M, Zwahlen M, Loddenkemper R (2008). Demographic characteristics of patients with extrapulmonary tuberculosis in Germany. Eur Respir J.

[21] Chhabra S, Kashyap A, Bhagat M (2021). Anemia and nutritional status in tuberculosis patients. Int J Appl Basic Med Res.

[22] Feleke BE, Feleke TE, Biadglegne F (2019). Nutritional status of tuberculosis patients, a comparative cross-sectional study. BMC Pulm Med.

[23] Guo X, Yang Y, Zhang B (2022). Nutrition and clinical manifestations of pulmonary tuberculosis: a cross-sectional study in Shandong province, China. Asia Pac J Clin Nutr.

[24] Piskur ZI, Mykolyshyn LI, COMORBIDITIES AT THE TUBERCULOSIS AMONG, CHILDREN (2021). Wiad Lek.

[25] Tuberculosis Prevention and Control Key Laboratory/Beijing Key Laboratory of New Techniques of Tuberculosis Diagnosis and Treatment /Institute for Tuberculosis Research/Department of Tuberculosis of the 8th Medical Center of Chinese PLA General Hospital (2022). Editorial Board of Chinese Journal of Antituberculosis. Expert consensus on the rational use of glucocorticoids in tuberculosis treatment. Chin J Antituberculosis.

[26] Ma Y, Qiu S, Zhou R (2022). Osteoporosis in patients with respiratory Diseases. Front Physiol.

[27] Hammami F, Koubaa M, Mejdoub Y (2021). The association between vitamin D deficiency and extrapulmonary tuberculosis: case-control study. Tuberculosis (Edinb).

[28] Nnoaham KE, Clarke A (2008). Low serum vitamin D levels and tuberculosis: a systematic review and meta-analysis. Int J Epidemiol.

[29] Shangguan X, Xiong J, Shi S (2022). Impact of the malnutrition on mortality in patients with osteoporosis: a Cohort Study from NHANES 2005–2010. Front Nutr.

[30] Byrne AL, Marais BJ, Mitnick CD (2015). Tuberculosis and chronic respiratory disease: a systematic review. Int J Infect Dis.

[31] Gomes T, Reis-Santos B, Bertolde A (2014). Epidemiology of extrapulmonary tuberculosis in Brazil: a hierarchical model. BMC Infect Dis.

[32] Lin JN, Lai CH, Chen YH (2009). Risk factors for extra-pulmonary tuberculosis compared to pulmonary tuberculosis. Int J Tuberc Lung Dis.

[33] Sreeramareddy CT, Panduru KV, Verma SC (2008). Comparison of pulmonary and extrapulmonary tuberculosis in Nepal- a hospital-based retrospective study. BMC Infect Dis.

[34] Alemu A, Bitew ZW, Diriba G (2021). Co-occurrence of tuberculosis and diabetes mellitus, and associated risk factors, in Ethiopia: a systematic review and meta-analysis. IJID Reg.

[35] Willson C, Watanabe M, Tsuji-Hosokawa A (2019). Pulmonary vascular dysfunction in metabolic syndrome. J Physiol.

[36] Ardigo D, Valtuena S, Zavaroni I, Delsignore R (2004). Pulmonary complications in diabetes mellitus: the role of glycemic control. Curr Drug Targets Inflamm Allergy.

[37] Tai H, Jiang XL, Yao SC et al. Vascular endothelial function as a valid predictor of variations in pulmonary function in T2DM patients without related complications. Front Endocrinol (Lausanne). 2021 Mar 11;12:622768. 10.3389/fendo.2021.622768 PMC7991996 33776922

[38] Ayelign B, Negash M, Genetu M et al. Immunological impacts of diabetes on the susceptibility of Mycobacterium tuberculosis. J Immunol Res 2019 Sep 9;2019:6196532. 10.1155/2019/6196532 PMC6754884 31583258

[39] Aslam MV, Owusu-Edusei K, Marks SM (2018). Number and cost of hospitalizations with principal and secondary diagnoses of tuberculosis, United States. Int J Tuberc Lung Dis.

[40] Ping Chu Y, Chang X, Zhang (2022). Epidemiology of extrapulmonary tuberculosis among pediatric inpatients in mainland China: a descriptive, multicenter study. Emerg Microbes Infect.

